# Interspecies Outer Membrane Vesicles (OMVs) Modulate the Sensitivity of Pathogenic Bacteria and Pathogenic Yeasts to Cationic Peptides and Serum Complement

**DOI:** 10.3390/ijms20225577

**Published:** 2019-11-08

**Authors:** Justyna Roszkowiak, Paweł Jajor, Grzegorz Guła, Jerzy Gubernator, Andrzej Żak, Zuzanna Drulis-Kawa, Daria Augustyniak

**Affiliations:** 1 Department of Pathogen Biology and Immunology, Institute of Genetics and Microbiology, University of Wroclaw, 51-148 Wroclaw, Poland; justyna.roszkowiak@uwr.edu.pl (J.R.); grzegorz.gula@uwr.edu.pl (G.G.); zuzanna.drulis-kawa@uwr.edu.pl (Z.D.-K.); 2 Department of Pharmacology and Toxicology, Faculty of Veterinary Medicine, Wroclaw University of Environmental and Life Sciences, 50-375 Wroclaw, Poland; pawel.jajor@upwr.edu.pl; 3 Department of Lipids and Liposomes, Faculty of Biotechnology, University of Wroclaw, 50-383 Wroclaw, Poland; jerzy.gubernator@uwr.edu.pl; 4 Ludwik Hirszfeld Institute of Immunology and Experimental Therapy, Polish Academy of Science, 53-114 Wroclaw, Poland; andrzej.zak@hirszfeld.pl

**Keywords:** outer membrane vesicles (OMVs), *Candida albicans*, antimicrobial peptides, complement, interspecies interactions, inter-kingdom protection, fungicidal activity, fluconazole, hyphae

## Abstract

The virulence of bacterial outer membrane vesicles (OMVs) contributes to innate microbial defense. Limited data report their role in interspecies reactions. There are no data about the relevance of OMVs in bacterial-yeast communication. We hypothesized that model *Moraxella catarrhalis* OMVs may orchestrate the susceptibility of pathogenic bacteria and yeasts to cationic peptides (polymyxin B) and serum complement. Using growth kinetic curve and time-kill assay we found that OMVs protect *Candida albicans* against polymyxin B-dependent fungicidal action in combination with fluconazole. We showed that OMVs preserve the virulent filamentous phenotype of yeasts in the presence of both antifungal drugs. We demonstrated that bacteria including *Haemophilus influenza*, *Acinetobacter baumannii,* and *Pseudomonas aeruginosa* coincubated with OMVs are protected against membrane targeting agents. The high susceptibility of OMV-associated bacteria to polymyxin B excluded the direct way of protection, suggesting rather the fusion mechanisms. High-performance liquid chromatography-ultraviolet spectroscopy (HPLC-UV) and zeta-potential measurement revealed a high sequestration capacity (up to 95%) of OMVs against model cationic peptide accompanied by an increase in surface electrical charge. We presented the first experimental evidence that bacterial OMVs by sequestering of cationic peptides may protect pathogenic yeast against combined action of antifungal drugs. Our findings identify OMVs as important inter-kingdom players.

## 1. Introduction

In the environment, different microbial populations, including bacteria and fungi, co-exist. Mixed microbial populations are also a common feature in many diseases. Hence, understanding which factors may be potentially important players in interspecies dynamics of growth and to what degree these dynamics are mediated by the host is very important. Among these factors are outer membrane vesicles (OMVs) of Gram-negative bacteria classified recently as secretion system type zero [1]. These proteoliposomal nanoparticles released from the cell play an important role in bacterial physiology and pathogenesis. During pathogenesis, they enhance cellular adherence, cause biofilm formation, and induce apoptosis or inflammation [2,3,4]. Furthermore, DNA-containing OMVs, through horizontal gene transfer, are important in interspecies communication as well as in host–pathogen interactions [5]. OMVs contribute also to innate bacterial defense through β-lactamase content or trapping and degrading the membrane-active peptides [6,7,8,9,10]. Accordingly, the involvement of OMVs in resistance to antibiotics with various modes of action is growing [11]. Both offensive and defensive functions of OMVs have been reported. In line with this, OMVs can deliver bactericidal toxins or enzymes to other bacteria. Lytic activities have been documented for OMVs derived from *Pseudomonas aeruginosa* or *Myxococcus xanthus* [12,13,14]. On the other hand, for example, *M. catarrhalis* OMVs carrying β-lactamases confer protection to *M. catarrhalis* and *Streptococcus pneumoniae* against β-lactam antibiotics [15]. Likewise, OMVs from β-lactam-resistant *E.coli* can protect β-lactam-susceptible *E.coli* and fully rescue them from β-lactam antibiotic-induced growth inhibition [16]. Several earlier studies have shown that OMVs are involved in the trapping of antimicrobials, thus providing protection among bacteria, essentially in intraspecies systems [6,8,17]. Nevertheless, limited data are available on the relevance of this phenomenon in various interspecies populations. Among mixed populations, the degree of OMV-dependent protection of individual partners can be completely different. One of the factors that may influence this is the physicochemical nature of the vesicle itself. It includes both the intrinsic antimicrobial binding capacity, which is the result of inherited or acquired resistance [18] but also the ability of vesicle, based on physicochemical properties, to interact with other target cells. There are several factors responsible for the latter phenomenon including electric charge and hydrophobicity of a cellular surface [19,20]. The metabolic activity of OMV-producing bacteria also seems to have a significant impact on this issue. For example, for *P. aeruginosa* it was reported that variation in physicochemical properties was dependent on growth phase (exponential versus stationary), influencing, therefore, the cell association activity [19]. All aforementioned characteristics of OMVs indicate the significant degree of selectivity in OMV–cell interactions. Therefore, considering variety of microbial populations, the question of which bacteria are and which are not protected against membrane-active agents is still very ambiguous. It is also of great importance to ask whether this protection can go beyond the protection against only bacteria, affecting pathogens from other kingdoms such as fungi. Hence, it is of interest to investigate the protective potency of OMVs in the light of their specificity to react with a target cell. In the present study, we used our well-characterized OMVs from *Moraxella catarrhalis* Mc6 [4,21] as model vesicles and characterized their protective potential against model membrane-active agents in various interspecies combinations. First, we examined the protective activity of OMVs in bacterial intra- and interspecies systems and mode of observed protection, showing by HPLC-UV, zeta potential measurement, and o-nitrophenyl-β-D-galactopyranoside ONPG-based permeabilization assay, highly effective sequestration. Next, we examined the protective potential of OMVs in the bacterial-yeast inter-kingdom system. As far as we know, our study is the first report that OMVs can protect *Candida albicans* against antifungals drugs. Finally, we investigated whether OMVs-dependent protection is a signature of only free vesicles or those associated with the cell.

## 2. Results

### 2.1. Mc6 OMVs Characteristics

**Figure 1:**
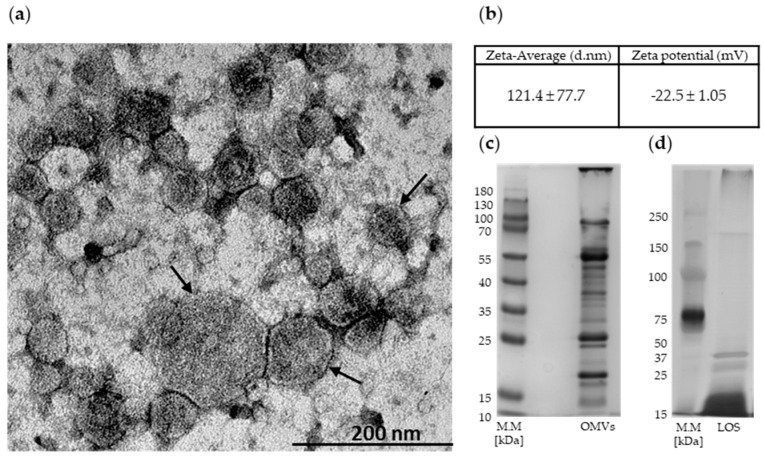
Physical characterization of outer membrane vesicles (OMVs) released from Mc6 cells: (**a**) TEM image of OMVs, vesicles are indicated by arrows (magnification, ×50,000); (**b**) the size distribution by volume and the zeta potential of vesicles, as assessed by the Zeta-sizer; each experiment was performed in triplicate; (**c**) the respresentative proteinogram of 12% SDS-PAGE electrophoresis of OMVs; the protein profiles were visualized using Coomassie staining; (**d**) the representative 10% SDS-PAGE electrophoresis of lipooligosaccharide (LOS)-OMVs visualized by silver staining.

As shown in transmission electron microscopy (TEM) image, the diameters of outer membrane vesicles from *M. catarrhalis* 6 had 30–200 nm (Figure 1a). The results of measurements of OMV particle size/zeta potential are shown in Figure 1b. The protein and lipooligosaccharide (LOS) components of OMVs are shown in Figure 1c,d, respectively. As we documented previously [21], the pivotal outer membrane proteins packaged in these vesicles were OmpCD, OmpE, UspA1 (ubiquitous surface protein A1, Hag/MID (*Moraxella* immunoglobulin D-binding protein), CopB, MhuA (hemoglobin-binding protein), TbpA (transferrin-binding protein A), TbpB (transferrin-binding protein B), LbpB (lactoferrin-binding protein B), OMP M35, and MipA (structural protein).

### 2.2. M. catarrhalis OMVs Passively Protect Cross-Pathogens against Polymyxin B-Dependent Killing

**Figure 2:**
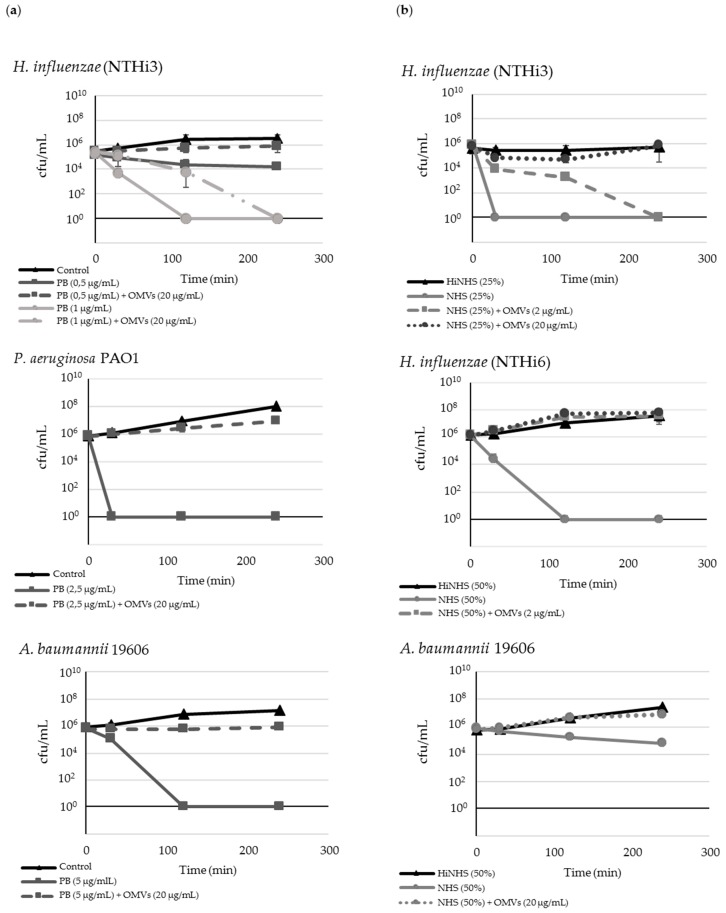
Mc6 OMVs protect bacteria from other species against polymxin B-dependent and human complement-dependent bactericidal activity: (**a**) Polymyxin B-dependent killing; (**b**) human serum (NHS) complement-dependent killing. Bacteria from log phase were incubated for 4 h in the presence of indicated concentrations of membrane-targeting agents alone or along with OMVs and plated in 0, 30, 120, and 240 min. Data are expressed as mean cfu/mL ± SD from three independent experiments performed in triplicate.

Polymyxin B (PB) was used as a model of cationic peptide. In our study, using 4-h time-kill assay, the minimal bactericidal concentrations (MBC) of PB against 5 × 10^5^–10^6^ cfu/mL of prominent human pathogens, including nontypeable *Haemophilus influenzae*, *Pseudomonas aeruginosa,* and *Acinetobacter baumannii*, was in the range 0.5–5 μg/mL, and caused killing effect within 2 h (Figure 2a). When pathogenic bacteria were incubated with a bactericidal concentration of PB in combination with 20 µg/mL OMVs from Mc6, the bacteria showed active growth in contrast to the antibiotic alone, which remained at the level of control. Up to 10 times lower concentrations of OMVs had no effect or the effect was negligible. The amount of OMVs that was required to achieve complete protection against respiratory pathogens referred to 20 µg/mL. The higher concentrations of vesicles did not intensify the growth.

### 2.3. OMVs Protect Serum-Sensitive Strains and Accelerate Growth of Serum-Resistant Strains against Complement

To define the influence of OMVs on the survival of cross-pathogens in the presence of active serum (NHS), the 4 h complement bactericidal assays were performed (Figure 2b). Of the three studied cross-pathogens, only nontypeable *H. influenzae* appeared to be serum-sensitive. Two others, *A. baumannii* and *P. aeruginosa,* were serum-resistant. The resistant strains showed either 100% survival or only a slight decrease (within one order) after 4 h of incubation in 50% or 75% NHS. Nontypeable *H. influenzae* strains were sensitive to NHS-dependent killing with a reduction of cfu/mL in the range of six orders after 30 min of incubation (NTHi3) or 120 min of incubation (NTHi6) in the presence of 25% or 50% serum, respectively, compared to the initial inoculum. These strains co-incubated with NHS and 20 μg/mL of OMVs exhibited a time-dependent increase in survival, which was similar to growth of control incubated in the presence of heat-inactivated serum (HiNHS).

*A. baumannii* incubated only in the presence of active NHS was slightly killed by the lytic complement action within 4 h, whereas after parallel exposure to OMVs, its growth was at the level of HiNHS control. *P. aeruginosa* was highly resistant to 75% action of serum, thus co-incubation with OMVs did not change the growth dynamics (data not shown). When control bacteria were incubated in the presence of heat-inactivated NHS, neither killing nor other growth alterations were observed for any of the tested strains, indicating that observed lysis was complement-dependent. Overall, these findings indicate that OMVs efficiently protect serum-sensitive gram-negative pathogens against complement action while accelerating the growth of moderately serum-resistant strains.

### 2.4. Mc 6 OMVs Passively Protect Pathogenic Yeasts against Polymyxin B-Dependent Fungicidal Effect in Combination with Fluconazole

**Figure 3:**
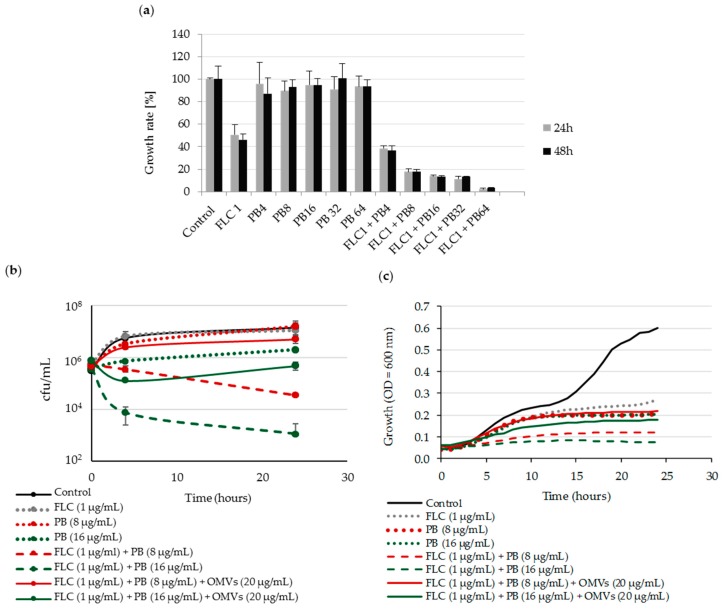
OMVs protect yeast against synergistic fungicidal activity of polymyxin B in combination with fluconazole: (**a**) Synergistic activities determined by checkerboard assay; (**b**) time-kill assay; (**c**) 24 h growth-curve kinetics for *C. albicans* incubated with drug alone and drug in combinations with or without OMVs. The kinetics were measured using Varioskan™ LUX reader with measurements at 1 h intervals. The data for experiments (a,b) show means  ±  SD from two independent experiments carried out in triplicate; the data for experiment (c) show mean values from two independent repetitions carried out in duplicate; for better readability, SDs were not included.

It has been previously reported that the antifungal effect of polymyxin B combined with fluconazole can be synergistic or potentiated [22]. We therefore initially examined the sensitivity of *Candida albicans* to combined action of both drugs. Our results showed, that for ~10^5^ cfu/mL of this yeast, the MIC_50_ for fluconazole (FLC) and MIC_100_ for polymyxin B (PB) were, respectively, 1 µg/mL and 128 µg/mL. Next, based on checkerboard assay, we assessed the growth of *C. albicans* incubated with polymyxin B alone, fluconazole alone, or their combinations at various concentrations. The results revealed that polymyxin B at concentrations much lower than MIC (1/8 MIC and 1/16 MIC) exerts a potent antifungal effect against *C. albicans* when combined with 1 µg/mL (MIC_50_) of FLC (Figure 3a).

Next, using time-kill assay we showed that after 24 h of incubation, the combinations of FLC^1^-PB^8^ and FLC^1^-PB^16^ (μg/mL) are fungicidal, which was expressed by one order and by two orders of decrease in viability, respectively (Figure 3b). In the presence of OMVs, this fungicidal action of both drugs was significantly abolished or weakened in comparison to action of a single drug and depending on PB concentration used. This protective effect of Mc6 OMVs against *C. albicans* was also confirmed in bacterial growth kinetics (Figure 3c). By estimating the profiles of *Candida* growth, as a result of every hour measurements carried out for 24 h at 37 °C, we have documented that the OMVs added to both aforementioned drug combinations abolished their inhibitory effect on *Candida* growth rate. Thus, the *Candida* growth in the presence of OMVs remained at the level for a single compound. Collectively, these results indicate that OMVs protect *C. albicans* from PB-dependent fungicidal effect in combination with fluconazole.

### 2.5. Mc6 OMVs Passively Enhance the Virulence of Pathogenic Yeasts Facilitating the Formation of Filaments

**Figure 4:**
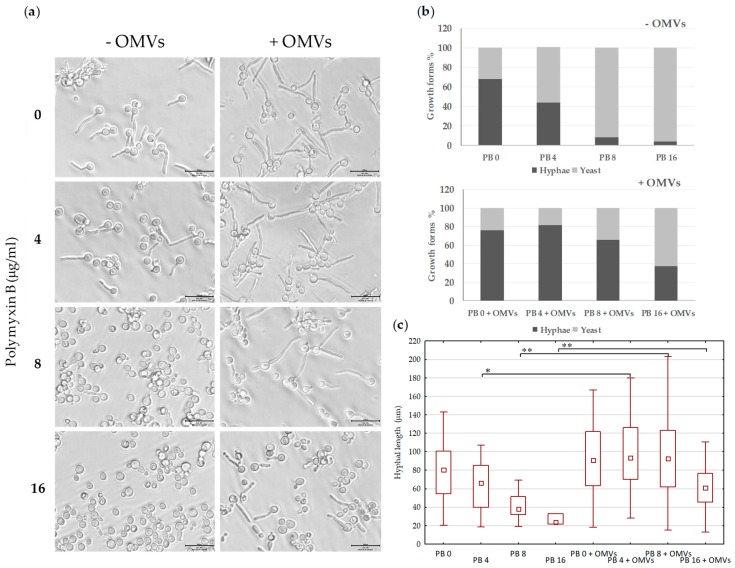
Effect of OMVs on filamentous growth of *C. albicans*: Yeast cells were preincubated overnight with 1/16 MIC of FLC (0.0625 µg/mL) without shaking and then, after washing, were incubated at OD = 0.4 in 0.4 mL of hyphae inducing medium (10% FBS in PBS) in 24-well microtiter plates with shaking (130 rpm) for 2 h at 37 °C in the presence of 0.0625 µg/mL FLC, indicated concentrations of PB, and with or without 20 µg/mL of OMVs. Three independent experiments were performed. (**a**) The filamentation was monitored under inverted microscope using 40× objective (Zeiss) and images were recorded. Scale bars = 100 µm. (**b**) The number of hyphal cells versus yeast cells was determined using ImageJ software. The pool of yeast cells contains yeasts and yeasts whose germ tubes did not exceed 10 µm. The data represent the mean values calculated for ≥ 200 cells for each of the eight tested options. (**c**) Box and Whisker plots of hyphal length: Minimal and maximal values, median, quartiles Q_1_ and Q_3_. Statistical analysis was performed by one-way ANOVA (* *p* < 0.05, ** *p* < 0.01).

It was documented that azole drugs are necessary both during growth and induction step to have an effect on transition yeast-to hyphae in *C. albicans* [23]. The presence of FLC in the decreased range between 1–0.125 μg/mL both during growth and induction step caused considerable inhibition of hyphal growth (data not shown). Therefore, to determine the effects of fluconazole in combination with polymyxin B on hyphal growth, the yeasts were initially preincubacted overnight at 37 °C with 1/16 MIC of FLC (0.0625 μg/mL). During the 2 h induction of hyphal growth in the presence of 10% fetal bovine serum, 1/16 MIC of FLC was added together with 1/8, 1/16, and 1/32 MIC of PB in the presence of absence of 20 µg/mL of OMVs. Figure 4a,b shows that the number of hyphal cells, shown by the ratio of cells in yeast form compared to that with formed filaments after 2 h of incubation, decreases as the concentration of polymyxin B increases. Under the tested conditions, the combinations FLC^0.0625^-PB^8^ and FLC^0.0625^-PB^16^ (µg/mL) showed almost complete inhibitory activity against the formation of hyphae. FLC^0.0625^-PB^4^ had also clear inhibitory effect on yeast-to-hyphae transition in comparison to FLC alone. The presence of 20 μg/mL OMVs in all tested drug combinations caused a significant increase in the percentage of hyphae-forming cells (Figure 4b), probably as a result of neutralizing PB-dependent potentiating effect of FLC. Furthermore, the increase in yeast-to-hyphal transition was accompanied by a significant extension of filaments (Figure 4c). In summary, these results indicate that in the presence of Mc6 OMVs, the inhibiting effect of FLC and PB, at concentrations significantly lower than MICs, on the formation of filaments is abolished. It shows that in the presence of OMVs, *C. albicans* may retain the virulent filamentous phenotype in the presence of both antifungal drugs, thus increasing its virulence.

### 2.6. OMV-Dependent Protection against PB and Complement is the Result of PB Sequestration on Free OMVs

**Figure 5:**
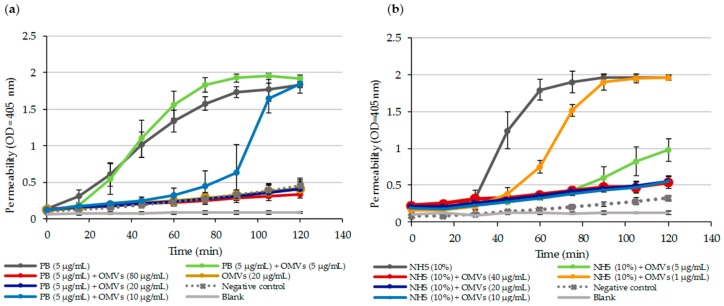
OMVs block the permeabilizing activity of membrane targeting agents. Change in *E. coli* ML-35p membrane permeability was assayed by a time-dependent influx of ONPG in the presence of membrane-targeting agents and different concentrations of Mc6 OMVs. Bacteria at log phase (~10^6^ cfu/mL) were incubated on microplate at 37 °C for 120 min in the presence of indicated concentrations of OMVs together with (**a**) polymyxin B (PB) and (**b**) normal human serum (NHS). The absorbance was measured at indicated time points. The results are shown as mean ± SD from at least three independent experiments performed in duplicate.

To study how efficiently PB is neutralized by Mc6 OMVs, various biological and biochemical methods were used. Initially, a decrease of free PB was confirmed indirectly using *E.coli* ML35p mutant with constitutive β-galactosidase expression (Figure 5a).

In the experiment, different concentrations of Mc6 OMVs were introduced into the system containing *E. coli* ML-35p mutant at a concentration of ~10^6^ cfu/mL and PB at a concentration of 5 µg/mL. By measuring the quantitative intracellular influx and hydrolysis of ONPG (β-galactosidase substrate), as a result of membrane permeabilization, we showed that OMV-dependent protection was dose-dependent and that PB was very quickly depleted from the environment in the presence of at least 20 µg/mL OMVs leading to a decrease in peptide activity (Figure 5a).

**Figure 6:**
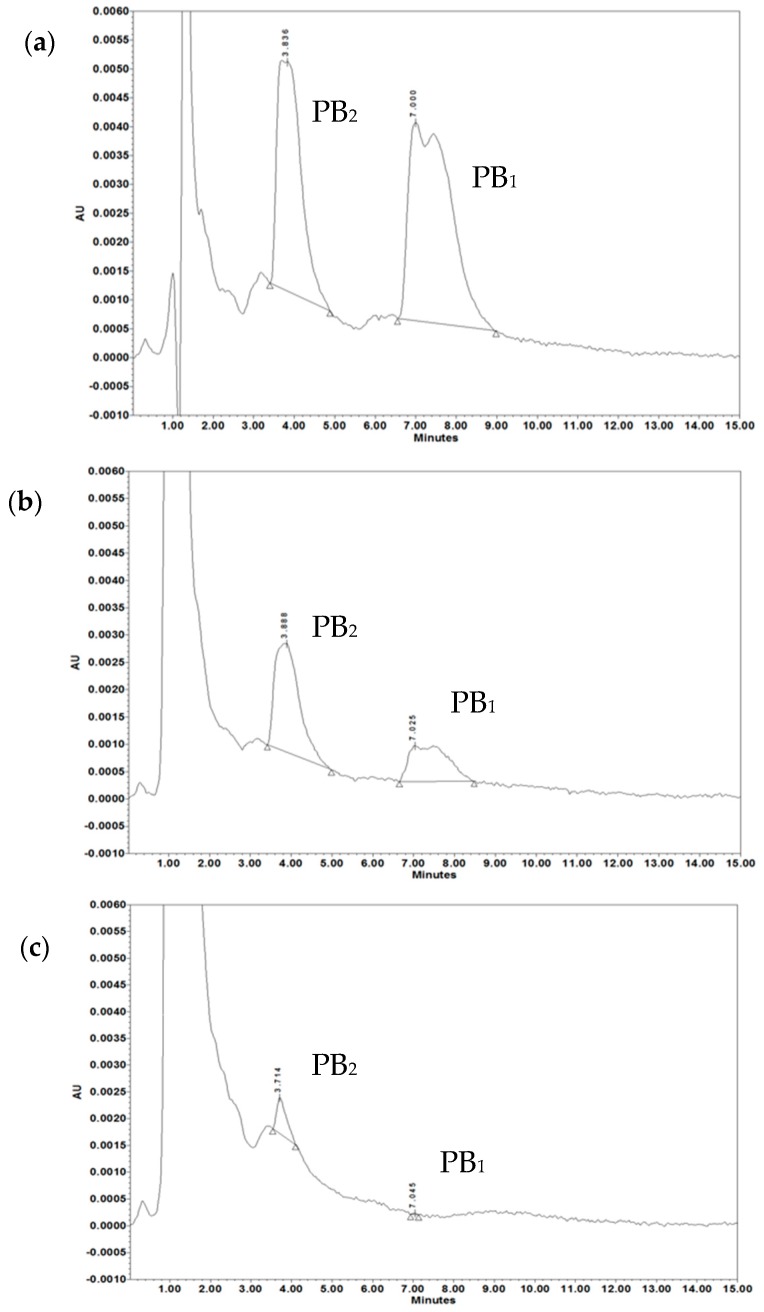
The HPLC spectra of polymyxin B incubated alone or along with OMVs. Chromatograms of (**a**) 50 µg/mL solution of polymyxin B, (**b**) 50 µg/mL of PB after treatment with 5 µg/mL of Mc6 OMVs, (**c**) 50 µg/mL of PB after treatment with 20 µg/mL of Mc6 OMVs. Samples were prepared as described in Materials and Method section. HPLC analyses were performed with Macherey–Nagel Nucleodur C18 Isis column. UV-detection at 215 nm. Double peak with Tr = 3.8 min corresponds mainly to polymyxin PB_2_ and PB_3_, and double peak with Tr= 7 min corresponds to polymyxin PB_1_ and PB_1_-I. The representative chromatograms are shown.

Next, to directly confirm the role of OMVs in PB sequestration, we determined zeta potential, showing that the initially moderately negative zeta potential of intact OMVs was immediately and significantly neutralized by addition of at least 50 µg/mL of PB, in a concentration-dependent and diluent-dependent manner leading to membrane depolarization (Table 1). The presented results of alteration in Zeta potentials were similar after 30 min and 60 min incubation at 37 °C (data not shown). Finally, to quantitatively assess the magnitude of PB sequestration by OMVs, the residual free PB content remaining after ultrafiltration of OMV-PB complexes was measured using HPLC-UV. Figure 6 shows the chromatograms of recovered by ultrafiltration PB that was preincubated for 30 min either alone or with OMVs. The loss of PB following incubation with OMVs at 5 µg/mL and 20 µg/mL was almost 60% (*p* < 0.001) and over 96% (*p* < 0.001), respectively, in reference to standard PB (Table 2).

Collectively, our results demonstrate that PB is quickly and effectively depleted from the environment via OMV-dependent sequestration.

Similar experiments with *E.coli* ML35p mutant were performed with human active serum (NHS). Similar to PB, the strong permeabilizing potency of membrane attack complex (MAC), present in 10% human serum, against *E.coli* ML35p was considerably decreased or even dumped as the concentration of OMVs increased (Figure 5b). It suggests that by deposition of complement components on the OMV surface, vesicles trigger MAC formation away from target bacteria and thus protect them from MAC-mediated lysis. Accordingly, based on the quantitative formation of soluble non-proteolytic membrane attack complex (SC5b-9), we demonstrated that the Mc6 OMVs activate human serum complement in a concentration-dependent manner (Figure S1).

### 2.7. OMVs Associated with Bacteria do not Protect against PB

**Figure 7:**
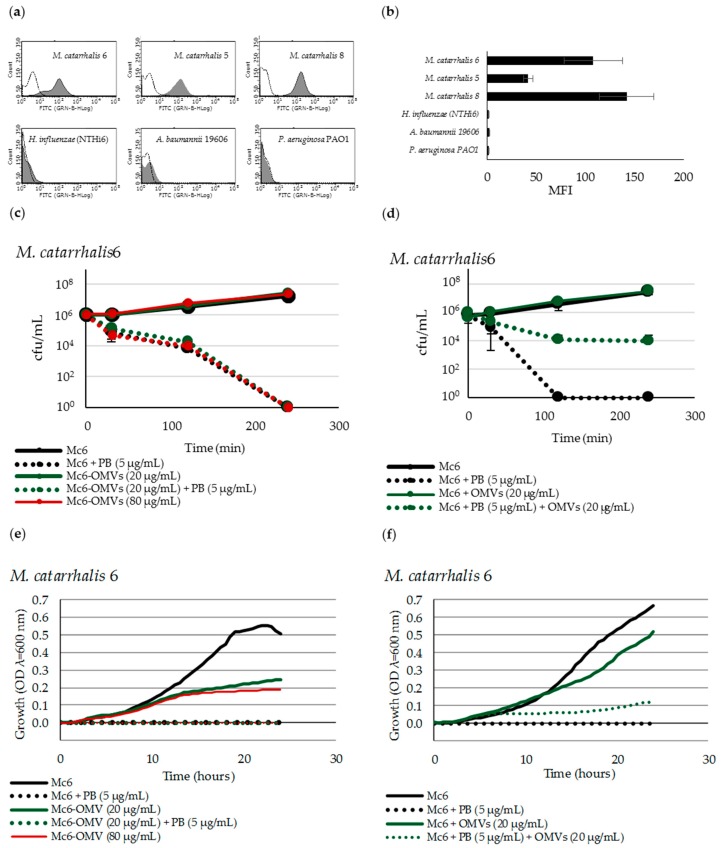
Mc6 OMVs highly associated with bacteria do not protect against polymyxin B-dependent killing. (**a**) Flow cytometry analysis of fluorescein isothiocyanate (FITC)-labelled OMVs associated with intraspecies bacteria (upper panel) and not associated with interspecies bacteria (lower panel), The fluorescence intensities of OMV-associated bacteria are shown as black histograms whereas control bacteria as dotted histograms. Representative plots are shown. (**b**) Quantification of bacteria associated with FITC-labelled OMVs. Data are expressed as mean fluorescent intensity (MFI) ± SD from three independent experiments performed in duplicates. (**c**) Bactericidal activity of polymyxin B against *M. catarrhalis* after association with OMVs. (**d**) Bactericidal activity of polymyxin B against *M. catarrhalis* incubated with free OMVs. (**e**) 24 h growth-curve kinetics for *M. catarrhalis* after association with OMVs. (**f**) 24 h growth-curve kinetics for *M. catarrhalis* incubated with free OMVs. The kinetics was measured using Varioskan™ LUX reader with measurements at 30 min intervals. In experiments (c,d) results are shown as mean ± SD from at least two independent experiments. In experiments (e,f) results are shown as mean from at least three independent experiments performed at duplicate and for better readability, SDs were not included.

To answer the question of whether Mc6 OMVs protect bacteria against the membrane-active agent by sequestration only indirectly, being far from the bacterial surface or through the direct shield of these cells, we determined the association capability of OMVs using flow cytometry. We used 4-times higher concentration of OMVs (80 μg/mL) to make sure that the entire cell population could be covered (Figure S2). We found that Mc6 OMVs incubated with studied strains strongly interacted within 30 min with the parental strain as well as with other strains from *Moraxella* species. After this time, more than 93% of measured events were fluorescent, indicating that FITC-labelled OMVs were associated with unlabeled bacteria. No increase in fluorescence was observed when Mc6 OMVs were incubated even up to 2 h with bacteria from other species indicating the complete lack of association (Figure 7a,b). These results indicate that OMVs released by *M. catarrhalis* associated only in intraspecies but not in interspecies tested systems, pointing to the specificity of this reaction. To verify whether the covering of *M. catarrhalis* with OMVs is another mode of protection against PB, 4 h time-kill assays (Figure 7c,d), as well as 24 h real-time bacterial growth kinetics (Figure 7e,f) were carried out on OMV-associated and not associated cells in the presence and absence of PB.

Not in the line with expectation, the results showed that for *M. catarrhalis* associated and not associated with OMVs, the lethal effect of PB occurred after 4 h (Figure 7c) and 2 h (Figure 7d), respectively. It indicates that the association does not ensure longer protection but only delays the bactericidal effect. The lack of protection was confirmed in growth kinetic experiments documenting that PB-dependent growth inhibition for OMV-associated *M. catarrhalis* and non-associated control was comparable and preserved for 24 h of incubation (Figure 7e). In the case of bacteria incubated in the presence of free OMVs and PB, the OMV-dependent protection is assured for ~7 h being on the level of control growth. Some modest and stable level of protection is maintained for 24 h (Figure 7f). Compared to the growth profiles of OMV-associated and non-associated *M. catarrhalis*, it was shown that the interaction with vesicles weakens the growth dynamics of the former. Overall, the results are evidence that OMVs associated with bacteria do not protect against PB, assuming of course that the bacterial cell is completely shielded by them. Consequently, it suggests the active fusion between OMVs and target cell membrane rather than only surface association.

## 3. Discussion

Bacteria and yeasts employ a variety of means to protect their envelopes against harmful environmental factors. Some of these factors cause membrane permeabilization, while others can inhibit the synthesis of cellular membrane components affecting the physical properties of the membrane [24,25]. The common strategy of bacteria to avoid membrane targeting factors is to overcome the negative charges of their surface envelope [24,26]. Alternatively, bacteria can release OMVs that act as extracellular decoys for some antimicrobials.

In this study, we report that OMVs may serve as a model of passive interspecies protection of prominent pathogenic bacteria but also pathogenic yeasts. While documenting the lethal effect, we showed that free OMVs block both the bactericidal action of peptide antibiotic polymyxin B and the lytic activity of complement. By quantitative measurement of alteration in electric charge on the OMV surface treated with PB as well as free PB determination by HPLC, we showed that 20 μg/mL of OMVs that had a significant protective effect against interspecies microorganisms caused almost complete depletion of this model cationic antimicrobial agent after co-incubation, thus indicating the immediate sequestration of the peptide on free OMVs. Accordingly, in similar experiments, we showed that OMVs inhibit serum lytic activity against prone pathogens, indicating that by deposition of complement components on the OMV surface, vesicles trigger MAC (SC5b-9) formation away from target bacteria and thus protect them from MAC-mediated lysis. The intensity of OMV-dependent complement activation in vitro was correlated with the number of free vesicles in the environment. Therefore, it is possible that any fluctuation in the number of released vesicles may positively or negatively influence complement activation of this potent innate mechanism.

Next, using the PB-dependent model, we investigated whether OMVs-dependent protection is a signature of only free vesicles or those associated with the cell. Using flow cytometry, we documented that the association of OMVs with bacterial cells may be very potent and species-specific, influencing the cell sensitivity to antimicrobial compound. Our vesicles were able to associate only with representatives of *M. catarrhalis* species while they did not work in interspecies systems. By evidencing the lethal effect of cell-associated OMVs exposed to polymyxin B, we showed that OMVs do not protect against AMPs this way, suggesting a fusion between OMVs and OM rather than only shielding. It is tempting to speculate that the specificity of interaction between OMVs and recipient cell is somehow pivotal in its sensitization at least to AMPs. We are therefore in agreement with Tashiro et al. that elucidating the selectivity in OMV-cell interaction is critical for an improved understanding of the outcome of this reaction. On the other hand, it has been documented that interactions with OMVs for some cross-pathogens can be specific, less specific, or not specific at all [27]. Thus, the OMV-dependent interplay may contribute to the generation of synergistic or antagonistic interactions between pathogens, resulting in more or less harmful outcomes for the host.

Next, we address the question of whether OMV-dependent trapping of cationic antimicrobials can protect pathogens from other kingdoms such as fungi of the species *C. albicans*. Polymyxins alone are effective against *C. albicans* only at relatively high concentrations [28]. Azoles including fluconazole are common antifungal drugs [29]. They affect the integrity of fungal membranes, altering their morphology and inhibiting growth [30,31]. It has been reported that quinolone and other antibiotics may augment the anti-candidal activity of azole and polyene agents [32]. Previous research also documented that PB in lower concentrations exerts a potent antifungal effect when combined with fluconazole [22,33]. Therefore, the elimination of the antibiotic from these systems by means of OMVs should decrease the fungus susceptibility to fluconazole. To test this hypothesis, we incubated *C. albicans* in the presence of both drugs and OMVs using FLC at MIC_50_ (1 µg/mL) and PB in the range much lower than MIC (1/8–1/16). To our knowledge, for the first time, we have provided evidence that bacterial OMVs can inhibit the fungicidal action of certain combined antifungal agents that are effective against pathogenic yeasts. Next, we documented that sub-MIC concentrations of FLC (1/16) and PB (in the range of 1/8–1/32) applied together weakened or almost completely inhibited the yeast-to-hyphae transition. Furthermore, in the presence of OMVs, the filamentous phenotype was considerably recovered and even strengthened despite exposure to both drugs. It is a very undesirable action of OMVs, since filamentation increases the virulence of *C. albicans,* which in hyphae form is more invasive and can attach in a higher number to epithelial cells than yeast and pseudohyphae forms [34,35]. Using analogies to the role of OMVs contributing to the sequestration of PB, it is tempting to speculate that the same mechanism of action exists in this case. Collectively, both of the aforementioned activities of bacterial vesicles seem to render *C. albicans* less vulnerable to destruction. The documented inter-kingdom OMV-based mutualistic relationship between bacteria and yeasts are, to our knowledge, a novel phenomenon. Therefore, its importance for more complex in vitro and in vivo conditions, as well as pathophysiology, remains to be clarified.

The complex *Candida*–bacteria interactions are not a rare occurrence and may have an important impact on the human disease by causing e.g., the faster biofilm growth or *Candida*-dependent induction of antimicrobial resistance of *Staphylococci* [36,37]. Sometimes, the results investigating cross-kingdom polymicrobial interactions are very contradictory even for the same microbial components. It was shown that direct or indirect (by released soluble molecules) contact of *P. aeruginosa* or *A. baumannii* with *C. albicans* or other fungi may lead to the killing of yeast [38,39]. It can also decrease fungal filamentation, biofilm formation, and conidia biomass [40]. The synergistic collaboration between the two pathogens was also documented. For example, the pre-colonization with *C. albicans,* which compromises the immune system, facilitates the emergence of *A. baumannii* pneumonia [41]. The interactions between microbes are not only affected by the specific combination of microorganisms, but also by the environment such as immunological milieu. Therefore, bacterial OMVs shape the behavior of neighboring microbes and the overall outcome of their interplay for the host. Due to the limited antifungal arsenal, the synergistic effect of PB and fluconazole or human broad-spectrum AMP lactoferrin with amphotericin B or fluconazole, which increases the activity of the antifungals against *Candida* spp, could be an alternative for treatment [22,42]. Likewise, AMPs such as defensins or gramicidin have shown to be a promising alternative to the current antimycotic and antibacterial therapies [43,44,45]. Furthermore, AMPs display a lower propensity to develop resistance than do conventional antibiotics [45]. In light of our research, however, these promising strategies should consider the unfavorable role of OMVs as a trap for host cationic peptides in mixed bacterial and bacterial–fungal infections. Our results may also have a meaning in medical microbiology. Because AMPs are already used in topical nasal antimicrobials in the treatment of nasal or paranasal cavity infections (sinusitis, maxillary, otitis media) [46,47], it is conceivable that abundantly produced OMVs, by very efficient AMP sequestration, may decrease the pharmacokinetics of these compounds.

Another important issue of our results is the number of OMVs needed to show biological activity. In general, the information about the number of OMVs produced in vivo is still very limited. Although OMV production in the course of infection has been documented [48], the magnitude of this production was not given. During the in vitro part of this study, however, the biologically active concentration of OMVs was 5 μg/mL per 10^3^ cfu/mL. In our study, 20 μg/mL of OMVs was protective against 10^6^ cfu/mL, indicating that OMV amounts used in our study are rational and may resemble the infectious condition.Furthermore, there is many data on how different stress factors may induce bacterial hypervesiculation incuding temperature stress [49], oxidative stress [50], hyperosmotic stress [49], or antibiotic stress [51]. So far, the correlation between OMVs production and pathophysiology of a specific disease was proven both in animal sepsis-like inflammation models [52,53,54] and in a patient with fatal meningococcal septicaemia [55]. Based on these aforementioned examples, the OMVs concentrations, which seems to be clinically relevant, are in the range 5–20 µg/mL.

Overall, our results on the protective and somehow deleterious for pathogens role of OMVs, in the bacteria–bacteria and bacteria–yeast interspecies systems, underline the enormous potential of these nanostructures as accelerating factors in case of various mixed infections. Furthermore, the OMV-dependent mode of actions may serve as a model of passive resistance of gram-negative bacteria not only to antimicrobials, but also to humoral defense components, which operate to disrupt cell membrane. Likewise, for dimorphic yeasts, the ability of OMVs to sequester membrane active compounds that augment the antifungal activity of azoles may have an important impact on *Candida* virulence. This work may serve as an important basis for further evaluation of OMVs-dependent interactions within pathogenic bacterial-fungal communities. Our results indicate that OMVs are important players in interspecies and cross-kingdom microbial interactions.

## 4. Materials and Methods

### 4.1. Materials

#### 4.1.1. Reagents

BHI (Brain Heart Infusion, OXOID, Basingstoke, UK) ); TSB (Tryptone Soya Broth, OXOID, Hampshire, England); TSA (Tryptone Soya Agar, OXOID, Hampshire, England), Bradford reagent (Protein Assay Dye Reagent Concentrate, Bio-Rad, München, Germany); β-nicotinoamide adenine dinucleotide hydrate (NAD, Sigma-Aldrich, Steinheim, Germany); fluconazole (FLC, Sigma-Aldrich, Poznan, Poland), hemin (Sigma-Aldrich, St. Louis, MO, USA); polymyxin B sulfate salt (PB, Sigma-Aldrich, Denmark); fluorescein isothiocyanate (FITC, ThermoScientific, Rockford, IL, USA); Hank’s Buffer with Ca^2+^, Mg^2+^ (HBSS, PAN Biotech, UK); RPMI 1640 (Lonza, Walkersville, MD, USA); *o*-nitrophenyl-β-D-galactopyranoside (ONPG, Sigma, Steinheim Germany), heat inactivated (56 °C, 1 h) FBS (Fetal bovine serum, Gibco Life Technologies, Grand Island, NY, USA).). HPLC chemicals: Acetonitrile (Sigma, München, Germany) for separation was HPLC far UV/gradient grade (J. T. Baker, Avantor™ Performance Material); 32 mM Na_2_SO_4_ solution for chromatographic usage was prepared with 4.5 g anhydrous sodium sulfate (POCh, Avantor™ Performance Material, Gliwice, Poland) and MiliQ (ultrapure water made with Simplicity UV Water Purification System, Merck Millipore, Saint-Quentin, France).

#### 4.1.2. Microbial Strains and Growth Condition

The following microbial strains were used: *Moraxella catarrhalis* (Mc5, Mc6, Mc8), nontypeable *Haemophilus influenzae* (NTHi3, NTHi6), *Acinetobacter baumannii* (ATCC 19606), *Pseudomonas aeruginosa* (PAO1), *Candida albicans* (Ca1), mutant of *Escherichia coli* ML-35p, a lactose permease-deficient strain with constitutive cytoplasmic β-galactosidase. All strains were from the collection of our Institute. *M. catarrhalis* strains were grown on Columbia agar plates or BHI broth. NTHi strains were grown on chocolate agar plates or in BHI broth supplemented with hemin and NAD at final concentrations of 15 μg/mL each. *Moraxella* and *Haemophilus* strains were cultivated at 37 °C with 5% CO_2_. *A. baumannii; E. coli ML-35p* and *P. aeruginosa* were routinely cultured in TSB medium at 37 °C. *C. albicans* was cultured in yeast extract-peptone-glucose (YPG) in 37 °C.

### 4.2. Methods

#### 4.2.1. Outer Membrane Vesicles Isolation

Outer membrane vesicles (OMVs) isolation was performed as described previously [21]. The protein concentartions of purified OMVs preparations was determined by Bradford assay) and the quality of OMVs preparation was confirmed in 12% SDS-PAGE.

#### 4.2.2. Time-Kill Assay

For testing PB or human serum (NHS) activity, the log-phase bacterial suspension (5 × 10^5^–10^6^ cfu/mL) was incubated with or without PB (in the range 0.5–5 µg/mL) or NHS (in the range 25%–75%) with the presence or absence of free OMVs (2 µg/mL or 20 µg/mL) in the final volume of 200 µl 1% medium (*w/v*). The experiments were performed from 0 to 240 min and the 10 µl aliquots of 10-time diluted bacterial suspensions were plated in triplicate on appropriate agar plates at 0, 30, 120, and 240 min time points. The colony counts and cfu/mL were calculated the next day. The controls for NHS contained heat-inactivated serum (HiNHS), (56 °C, 30 min). The bactericidal activity of PB or NHS was expressed in each time point as cfu/mL in reference to cfu/mL in time 0. Analogous experiments with bacteria associated and non-associated with OMVs (20 µg/mL or 80 µg/mL) in the presence of PB (5 µg/mL) were performed. Analogous experiments for selected combinations of antifungals were carried out for *C. albicans* except that: (i) Initial inoculum was ~2 × 10^5^–5 × 10^5^ cfu/mL, (ii) tested antimicrobials were PB (8 µg/mL or 16 µg/mL) and fluconazole (1 µg/mL), (iii) the medium was 0.5% YPG (*w/v*), and (iv) incubation lasts 24 h. All microbicidal assays were performed at least two times in triplicate.

#### 4.2.3. Growth Kinetics Assay

All growth kinetics experiments were performed on the flat-bottomed 96-well microplates (NUNC, Denmark) at 37 °C in volume 200 µL. Dynamic of growth was measured using Varioskan™ LUX reader with measurements at 30 min intervals for bacteria at 10^6^ cfu/mL diluted (final concentration) in 1% BHI (*w/v*) and 60 min intervals for yeast at 10^5^ cfu/mL (final concentration.) diluted in 0.5% YPG.

OMV-associated bacteria preparation: 0.5 mL of ~10^8^ cfu/mL exponentially growing bacteria were washed by centrifugation with HBSS Ca^2+^ Mg^2+^ and resuspended in 100 µl OMVs (20 µg/mL or 80 µg/mL) in Eppendorf tube. Association was performed for 30 min at 37 °C with gentle mixing. Thereafter, all samples were washed with HBSS Ca^2+^, Mg^2^ by centrifugation (8000 rpm, 10 min, 4 °C) to remove free OMVs particles, diluted to 2 × 10^6^ cfu/mL with HBSS Ca^2+^, Mg^2^, and used in growth kinetics assay.

#### 4.2.4. Checkerboard Microdilution Assay

The microdilution assay was performed on flat-bottom microplate according to the CLSI (formerly NCCLS) standard [56] except that the initial inoculum for *C. albicans* was ~10^5^ cfu/mL and cells were incubated at 37 °C without shaking. Synergy/growth potentiation was tested by the checkerboard method including a two-dimensional array of serial concentrations of both drugs. The fluconazole was used in concentrations 1–64 μg/mL whereas polymyxin B in concentrations 1–128 μg/mL. Wells without drugs or yeast inoculation were included as positive and negative controls, respectively. The MIC_100_ of polymyxin B and the MIC_50_ of fluconazole was defined as the lowest drug concentration that caused a decrease in absorbance of 100% and 50%, respectively, compared to control in drug-free medium.

#### 4.2.5. Induction of Filamentation

Before the induction of filamentation, *C. albicans* cells were preincubated overnight in YPG supplemented with sub-MIC concentration of FLC at 37 °C without shaking. To induce hyphal transition, the suspensions of *C. albicans* (OD = 0.4) were treated with 10% heat inactivated FBS in PBS for 2 h at 37 °C in 24-well flat-bottom microplates (NUNC) in volume 0.4 mL, in the presence of sub-MIC concentrations of FLC (1/16) and PB (range 1/8–1/32) and with shaking (130 rpm). The samples were observed under inverted microscope Zeiss Axio Vert. A1 with objective Zeiss LD A-Plan (40 × /0.55 Ph1). The images wer recorded using Industrial Digital Camera 5.1 MP 1 /2.5”. The assessment of cell morphology and the length (μm) of hyphae was performed using ImageJ software.

#### 4.2.6. Membrane Permeability Assay with ONPG

To assess the polymyxin B sequestration by *M. catarrhalis* OMVs in vitro, the time-dependent decrease of permeabilizing activity of this peptide against *E. coli* ML-35p with constitutive β-galactosidase activity was measured using the ONPG-mediated β-galactosidase microplate assay as previously described [57]. The final bacterial suspension (~10^6^ cfu/mL) in 10 mM sodium phosphate NaPB (pH 7.4) were incubated in the flat-bottom 96-well microplates (NUNC, Denmark) with 5 µg/mL of PB in the presence of OMVs at concentration range 1–20 µg/mL and with 3 mM ONPG as β-galactosidase substrate in the final volume of 150 μL. Microplates were incubated at 37 °C for 1.5 h and optical densities at λ = 405 nm were measured every 15 min (spectrophotometer ASYS). All the assays were performed at least 3 times in duplicates.

#### 4.2.7. Complement Activation

This method was described previously [58]. To assess complement activation by *M. catarrhalis* OMVs in vitro, the various concentrations of OMVs (1–20 μg/mL) were pretreated with active normal human serum (NHS) at a volume ratio 1:9 and the soluble terminal complement complex SC5b-9 was measured using ELISA kit according to manufacturer’s instructions (Quidel Corporation, San Diego, USA). Unstimulated NHS served as negative controls.

#### 4.2.8. OMV Association Assay and Flow Cytometry

OMVs labeling: Initially, OMVs (80 µg/mL) in PBS were concentrated using 50 kDa Vivaspin centrifugal concentrators (Amicon ultra, Millipore) at 14,000 × *g* for 10 min at 4 °C to remove PBS. The collected OMVs were reconstituted with 500 μL of 0.05 M carbonate/bicarbonate buffer (pH 9.5) and washed by centrifugation on vivaspin as described before. The collected OMVs at concentration 80 µg/mL were labeled with 1 mg/mL FITC at carbonate/bicarbonate buffer for 30 min at 37 °C with gentle mixing in the dark. The remaining fluorochrome was rinsed of 3 times with a 500 µL of cold carbonate/biscarbonate buffer each time, using 50 kDa Vivaspin. The final FITC-labelled OMVs were resuspended in Hank’s buffer Ca^2+^, Mg^2+^ at original concentration.

OMVs association with bacteria: 1 mL of each fresh bacterial culture corresponding to OD_550_ = 0.2 was centrifuged and subsequently washed with 1 mL of HBSS Ca^2+^, Mg^2+^ (8000× *g*, 10 min, 4 °C). The pellet was resuspended in 200 µL of OMV-FITC conjugate (80 µg/mL OMVs) and incubated for 30 min at 37 °C with gentle mixing in the dark. Afterward, samples were washed with HBSS Ca^2+^Mg^2^ by centrifugation (8000× *g*, 10 min, 4 °C) to remove free OMVs particles and finally resuspended in 200 µL of HBSS Ca^2+^ Mg^2^.

Flow cytometric analysis: To detect bacterial cells associated with FITC-labeled OMVs flow cytometry analysis was performed using GUAVA^®^ easyCyte flow cytometer (Millipore, Seattle, WA, USA). Before analysis, the samples were diluted 1:100 to obtain approximately 1–5 × 10^5^ cell/mL in HBSS Ca^2+^ Mg^2+^. Fluorescence intensity of bacterial cells associated with OMVs was analyzed for green fluorescence in the FL1 channel, by collecting 5000 events. Data were expressed as mean fluorescence intensity (MFI). Data analysis was performed using InCyte Merck Guava software (Millipore, Hayward, CA, USA).

#### 4.2.9. Estimation of Zeta Potential

The Zeta potential of OMVs was measured at room temperature (25 °C) by a Zetasizer Nano-ZS 90 (Malvern, UK). The instrument was equipped with a Helium–Neon laser (633 nm) as a source of light. The detection angle of Zetasizer at aqueous media was 173°. Considering the influence of factors such as conductivity (salt concentration) or pH of the solution on the Zeta potential, to minimize their influence, all Zeta potential measurements were performed with 10 mM NaPB buffer (pH 7.4) or in MiliQ.

#### 4.2.10. HPLC-UV System and Method

HPLC chromatography of polymyxin B was performed as described previously [26,59]. The HPLC system consisted of a Water’s 2695 Solvent Manager System with a built-in autosampler and 100 µL sample loop connected to Waters 2996 Photodiode Array Detector. Data collection and peaks integration were realized by computer with Water’s Empower 3 Chromatography Data Software. The separation developed with the use of a Macherey-Nagel EC Nucleodur C18 Isis column (50 mm, 4.6 mm ID with 1.8 µm beads) with Hypersil Gold aQ 5 µm (10 mm × 4.6 mm ID) guard precolumn. The mobile phase consisted of 22% acetonitrile and 78% of 32 mM Na_2_SO_4_ in water (pH 3.2 achieved with H_2_SO_4_) in isocratic separation mode. Eluent flow 0.75 mL/min and detection realized with a UV detector at 215 nm. Column and sample temperature were respectively 30 °C and 5 °C; separation run time was 15 min. The injection volume was 50 µL. The calibration curve was in the range of 1.56 µg/mL to 100 µg/mL prepared with 10 mg/mL standard stock solution of polymyxin B sulfate salt. Each concentration was injected twice daily for precision into 3 or 2 independent samples, and between day eight samples. The linearity of the method was in a range from 6.25 to 100 µg/mL with 16% of the average coefficient of variation (CV%) for the sum of peaks, and with a coefficient of determination R^2^ = 0.997. Since the major constituents of polymyxin B are B_1_ and B_2_ [59], (polymyxin B sulfate certificate of analysis, Sigma-Aldrich), these two components were analyzed altogether.

To quantify the results, the relative concentration was calculated as the mean PB concentration of sample/mean PB of control × 100%. Results were expressed as mean ± SD.

Sample preparation: 200 µL of reaction mixtures containing OMVs (5 µg/mL or 20 µg/mL) and PB (50 µg/mL) or PB alone (50 µg/mL) were incubated for 30 min at 37 °C with gentle mixing. The samples were ultrafiltrated using 50 kDa vivaspin centrifugal concentrators (Amicon ultra, Merck Millipore, Cork, Ireland) at 14 000× *g* for 15 min at RT to remove OMVs. The filtrate was collected and stored at −20 °C until use. Tested and control samples were processed identically.

#### 4.2.11. LOS-OMVs Electrophoresis

The concentration of lipooligosaccharide (LOS) from Mc6 OMVs was determined based on the purpald assay [60]. Diluted samples were solubilized in Laemmli sample buffer and heated at 100 °C for 5 min. Proteinase K (20 mg mL^−1^) was added per 20 mg of OMV proteins and incubated in a heating block at 60°C for 1 h. The presence of LOS in proteinase K-treated OMV samples was analyzed by dodecylosulfate gel electrophoresis. The 15 µL of samples were applied to the Glycine-SDS-PAGE (10%) gel path, corresponding to 5 µg LOS. Electrophoresis was carried during 2.5 h at a constant voltage of 80 V at 4 °C. The gel was fixed for 1 h at room temperature using a fixing solution (EtOH: Acetic Acid: MiliQ: 40:5:55); POCH, Gliwice, Poland) The fixed gel was stored overnight at 4 °C. MiliQ was exchanged for fresh before visualization using a modified Tsai and Frasch [61] silver staining protocol.

#### 4.2.12. TEM

The OMVs preparation for TEM was described previously [4]. OMVs were visualized by standard negative staining using a formvar copper grid (Christine Gröpl Electronenmikroskopie, Tulln, Austria) and 2% (*w/v*) aqueous solution of uranyl acetate. The OMVs were imaged with a TEM operating at an acceleration voltage of 150 kV (Hitachi H-800, Japan).

#### 4.2.13. Statistical Analysis

The data were expressed as the mean ± SD, and analyzed for the significant difference by one-way ANOVA or Kruskal–Wallis ANOVA rang using the Statistica (version 13.1) software (StatSoft, Krakow, Poland) Differences were considered statistically significant if *p* < 0.05.

## Figures and Tables

**Table 1:** Alteration in Zeta potential of OMVs treated with polymyxin B.

PB Binding by OMVs in NaPB Buffer		PB Binding by OMVs in miliQ	
Treatment	Zeta Potential (mV)	Treatment	Zeta Potential (mV)
20 µg/mL OMVs (control)	−22.5 ± 1.05	20 µg/mL OMVs (control)	−24.7 ± 0.76
20 µg/mL OMVs + 5 µg/mL PB	−21.2 ± 1.06	20 µg/mL OMVs + 5 µg/mL PB	−25.9 ± 0.97
20 µg/mL OMVs + 50 µg/mL PB	−16.5 ± 0.76 *	20 µg/mL OMVs + 50 µg/mL PB	−9.6 ± 0.35 *
20 µg/mL OMVs + 250 µg/mL PB	−10.1 ± 0.46 *	20 µg/mL OMVs + 250 µg/mL PB	−1.17 ± 0.14 *

*-*p* < 0.001 in reference to control as determined by one-way ANOVA.

**Table 2:** Magnitude of PB sequestration on *M. catarrhalis* OMVs.

Treatment	PB_2_ (µg/mL)	PB_1_ (µg/mL)	PB_2_ + PB_1_ (µg/mL)
Standard PB	14.38 ± 0.48	17.45 ± 3.58	31.83 ± 3.47
PB after treatment with 5 µg/mL OMVs	7.76 ± 1.01	5.19 ± 1.05	13.67 ± 1.33 *
PB after treatment with 20 µg/mL OMVs	0.96 ± 0.42	0.06 ± 0.01	1.02 ± 0.42 *

The PB content was measured by HPLC-UV method as described in material and methods. The results are expressed as mean ± SD. * *p* < 0.001 in reference to standard PB as determined by one-way ANOVA.

## Supplementary Materials

Supplementary materials can be found at https://www.mdpi.com/1422-0067/20/22/5577/s1.

## Abbreviations

AMP	Antimicrobial peptide
FBS	Fetal bovine serum
FLC	Fluconazole
HiNHS	Heat-inactivated normal human serum
LOS	Lipooligosaccharide
MIC	Minimal inhibitory concentration
NHS	Normal human serum
OMV	Outer membrane vesicle
ONPG	o-nitrophenyl-β-D-galactopyranoside
PB	Polymyxin B
