# Supplementary material for: Interspecies Outer Membrane Vesicles (OMVs) Modulate the Sensitivity of Pathogenic Bacteria and Pathogenic Yeasts to Cationic Peptides and Serum Complement
Source: Int J Mol Sci. 2019 Nov 8;20(22):5577. doi: 10.3390/ijms20225577 (PMC6888958; doi:10.3390/ijms20225577)
Supplement: Supplementary file 1 [file ijms-20-05577-s001.pdf]

Fig. S1

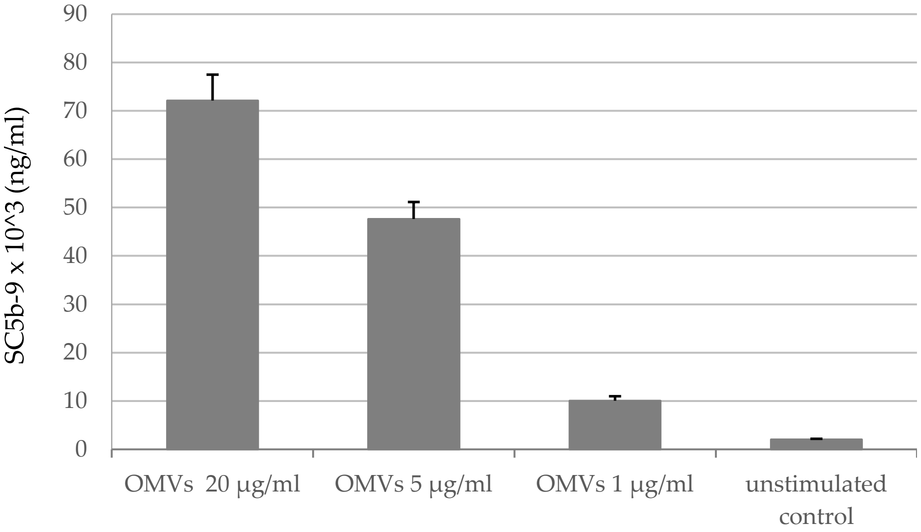

Figure S1. Human serum complement system activation by OMVs. The activation of the soluble non-proteolytic membrane attack complex (SC5b-9) was determined by ELISA. Before ELISA the OMVs (1-20 μg/ml) in veronal-buffer saline containing 0.15 mM Ca^{2+} and 0.5 mM Mg^{2+}, pH 7.4 were incubated with active human serum from healthy volunteers at a volume ratio of 1:9 at 37°C for 30 min with gentle agitation. Data are expressed as the mean ± SD of three independent experiments performed in duplicates. Statistics was performed by Kruskal-Wallis ANOVA, $*p < 0.001$ compared to unstimulated control.

Fig. S2

M. catarrhalis 6

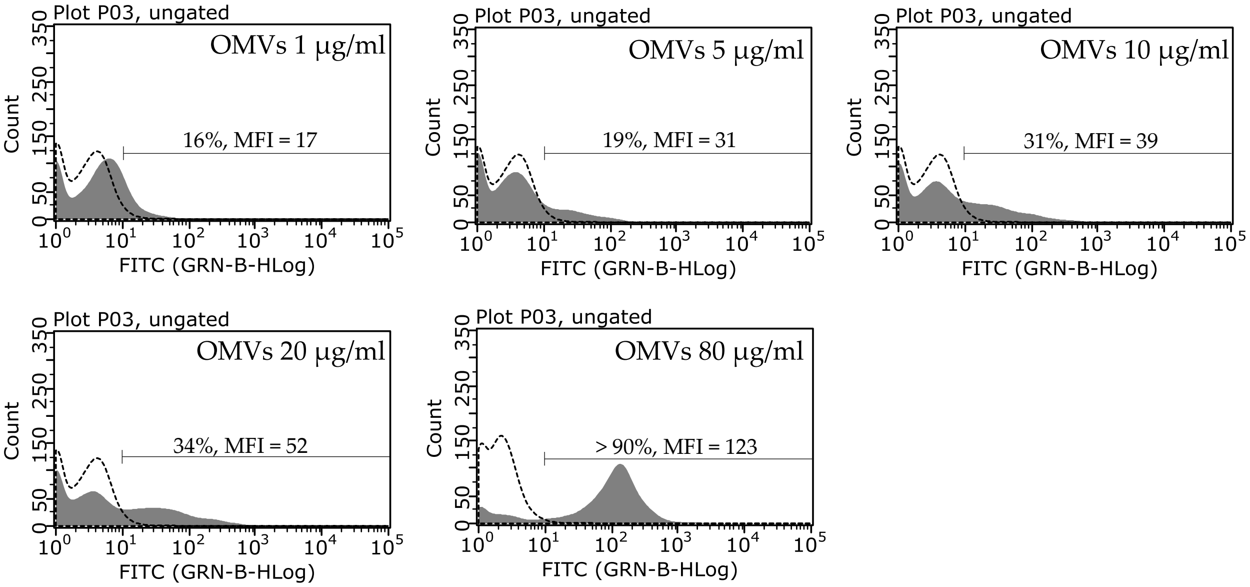

Figure S2. Flow cytometry analysis of FITC-labelled OMVs Mc6 associated with *M. catarrhalis* 6 in concentration-dependent manner. The fluorescence intensities of OMV-associated bacteria are shown as black histograms whereas control bacteria as dotted histograms. Quantification of bacteria associated with FITC-labelled OMVs is expressed as an events percentage and as mean fluorescent intensity (MFI).
